# Process analysis of the patient pathway for automated data collection: an exemplar using pituitary surgery

**DOI:** 10.3389/fendo.2023.1188870

**Published:** 2024-01-12

**Authors:** John G. Hanrahan, Alexander W. Carter, Danyal Z. Khan, Jonathan P. Funnell, Simon C. Williams, Neil L. Dorward, Stephanie E. Baldeweg, Hani J. Marcus

**Affiliations:** ^1^ Department of Neurosurgery, National Hospital for Neurology and Neurosurgery, London, United Kingdom; ^2^ Wellcome/EPSRC Centre for Interventional and Surgical Sciences, University College London, London, United Kingdom; ^3^ Department of Health Policy, London School of Economics & Political Science, London, United Kingdom; ^4^ Department of Neurosurgery, St Georges Hospital, London, United Kingdom; ^5^ Department of Diabetes & Endocrinology, University College London Hospitals National Health Service (NHS) Foundation Trust, London, United Kingdom; ^6^ Centre for Obesity and Metabolism, Department of Experimental and Translational Medicine, Division of Medicine, University College London, London, United Kingdom

**Keywords:** pituitary adenoma, patient pathway, process mapping, data collection, pituitary surgery

## Abstract

**Introduction:**

Automation of routine clinical data shows promise in relieving health systems of the burden associated with manual data collection. Identifying consistent points of documentation in the electronic health record (EHR) provides salient targets to improve data entry quality. Using our pituitary surgery service as an exemplar, we aimed to demonstrate how process mapping can be used to identify reliable areas of documentation in the patient pathway to target structured data entry interventions.

**Materials and methods:**

This mixed methods study was conducted in the largest pituitary centre in the UK. Purposive snowball sampling identified frontline stakeholders for process mapping to produce a patient pathway. The final patient pathway was subsequently validated against a real-world dataset of 50 patients who underwent surgery for pituitary adenoma. Events were categorized by frequency and mapped to the patient pathway to determine critical data points.

**Results:**

Eighteen stakeholders encompassing all members of the multidisciplinary team (MDT) were consulted for process mapping. The commonest events recorded were neurosurgical ward round entries (N = 212, 14.7%), pituitary clinical nurse specialist (CNS) ward round entries (N = 88, 6.12%) and pituitary MDT treatment decisions (N = 88, 6.12%) representing critical data points. Operation notes and neurosurgical ward round entries were present for every patient. 43/44 (97.7%) had a pre-operative pituitary MDT entry, pre-operative clinic letter, a post-operative clinic letter, an admission clerking entry, a discharge summary, and a post-operative histopathology pituitary multidisciplinary (MDT) team entries.

**Conclusion:**

This is the first study to produce a validated patient pathway of patients undergoing pituitary surgery, serving as a comparison to optimise this patient pathway. We have identified salient targets for structured data entry interventions, including mandatory datapoints seen in every admission and have also identified areas to improve documentation adherence, both of which support movement towards automation.

## 1 Introduction

Pituitary adenomas are intracranial tumours that are burdensome on patients and health systems due to their effects on quality of life (QoL) (1–3). Their endocrine and ophthalmic consequences can be severe, which is a driving force for research efforts looking to improve healthcare delivery for this patient group. Whilst amongst the commonest primary intracranial tumours, they are a relatively rare pathology with some subtypes meeting the criteria for rare diseases in the UK (4). Studying this patient group can therefore be challenging.

Digital healthcare, including electronic health records (EHRs) (5) and advancements in big data and artificial intelligence (AI) (6), has revolutionized pituitary adenoma research (7). However, the challenge lies in obtaining high-quality data, often requiring manual extraction, which is both labor-intensive and costly (8) (9). There is evidence that automated data collection is more accurate in some circumstances compared to manual data collection (8–10) and evidence that it can be cost saving (11). But poor data quality renders automation inaccurate and ineffective, which limits the value of any following activity using this data. Achieving high quality data and data collection infrastructure are recognised challenges facing health systems (12), meaning interventions to improve inputs and processes of data systems are a high priority. Improving data entry, therefore, would assist the accuracy of automation (13, 14). Structured data entry is one method of improving data accuracy (15, 16) which has been used to support data collection and analysis of EHRs (17, 18).

Pituitary patients are managed in a multi-disciplinary fashion and thus are a prime example of care delivered by a combination of medical and surgical specialities, offering rich datasets to study this patient group and their management. Patient pathway research across the pituitary MDT has shown to improve safety (19), however, this multidisciplinary care involves a range of users generating data, resulting in a heterogeneous dataset spread across multiple aspects of the EHR. This can make it challenging to identify and extract data for audit or research manually and is a barrier to automating collection of such data (9).

Yet, the EHR as a documentation tool provides a record of critical patient events as clinicians have a duty to accurately document such events. Whilst data entry is heterogeneous, there are activities that are consistently documented for particular patient groups, which are often required for a patient’s care to take place (e.g. an operation note for a patient undergoing surgery). These activities provide salient targets to improve the entry of data into the EHR to reduce data heterogeneity and structure it in a fashion amenable to automated extraction. To achieve this, the optimal areas of documentation to target must be identified and then validated to ensure they are reliably present in the EHR.

Process mapping is a system engineering methodology that provides a “current state” of a system, proven to be an effective tool in mapping patient pathways (20–22). Process mapping is particularly useful in depicting complex processes with multiple stakeholders, to provide a shared understanding of a process amongst stakeholders; an understanding critical in deciding how best to target quality improvement interventions. We hypothesize that this technique can be used to accurately map the patient pathway, using pituitary adenoma patients undergoing surgery as an exemplar. Validating the process map using real world data will help identify consistent and reliable documentation items to target to improve data entry into the system.

This study aimed to analyse the patient pathway of patients with pituitary adenoma undergoing surgical resection, producing a validated patient pathway. This would allow identification of the most reliable areas in the patient pathway to target structured data entry interventions.

## 2 Materials and methods

### 2.1 Study design

**Figure 1:**
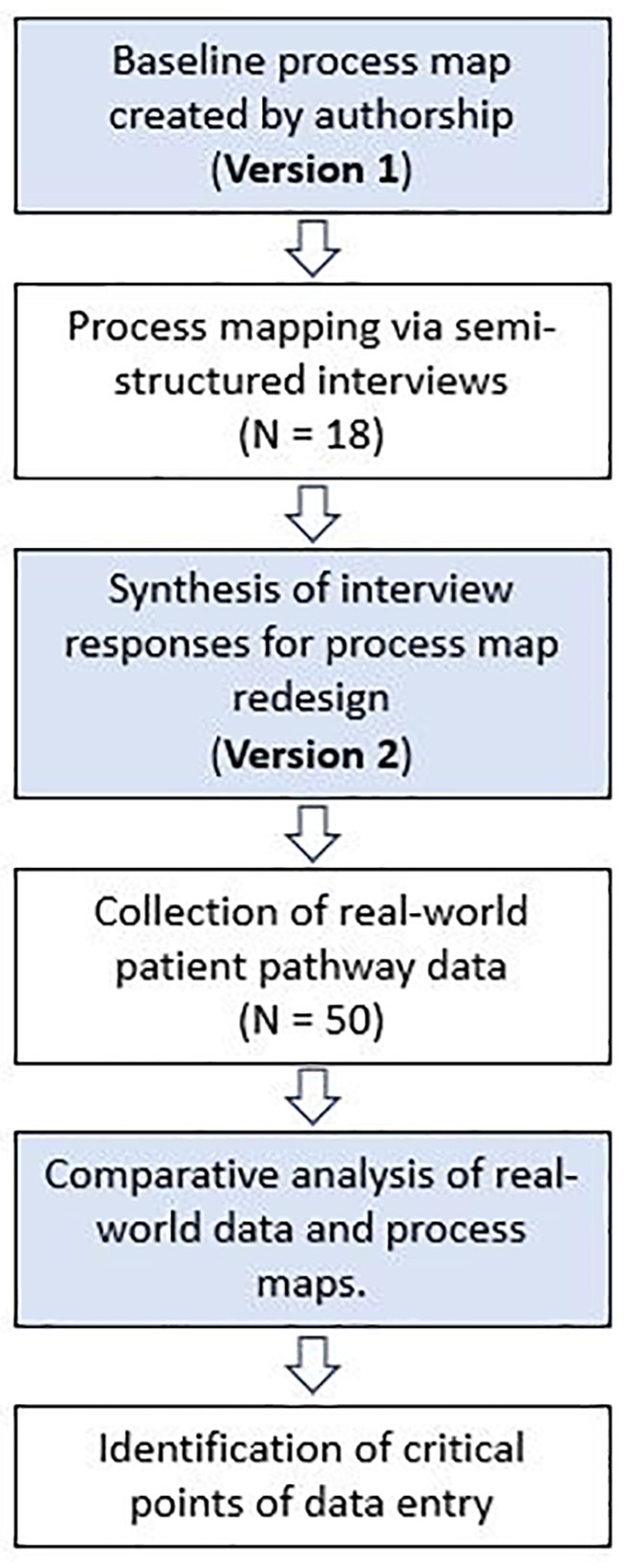
Study flow diagram.

This is a two-stage, mixed-methods study incorporating qualitative and quantitative methods (**Figure 1**) conducted between October 2021 and November 2021. This project was approved locally by University College London Hospitals trust as a service evaluation project. The population studied included patients undergoing surgery for a pituitary tumour in the largest pituitary centre in the UK. The process analyzed was the patient pathway for patients with a pituitary tumour undergoing surgical resection, beginning from referral to our centre and finishing at discharge from neurosurgical follow-up. Prospectively collected qualitative data was extracted from stakeholders through semi-structured interviews. The framework proposed by Antonacci et al. for process mapping phases was used to inform the methodology (20). Initial stakeholders were identified through production of the current state (Version 1) process map developed by the first (JGH) and senior author (HJM). Stakeholders were defined as individuals directly involved in the pathway of patients undergoing surgery for resection of a pituitary adenoma. A purposive snowball sampling approach was employed during process mapping sessions (23), revealing additional stakeholders from discussion with MDT members. Saturation was achieved when no new stakeholders were identified. A five-item questionnaire was produced to elicit baseline characteristics, perceptions and initial feedback from participants involved in developing Version 2 of the current state process map (**Appendix A**).

### 2.2 Process map development

All versions of the process maps were created using an online software (Lucid Chart, Lucid Software Inc, US). Version 1 was devised by two authors (JGH and HJM) based upon clinical experience. Semi-structured interviews were then arranged with process stakeholders on an individual basis. Participants completed the questionnaire (**Appendix A**) and reviewed the information sheet prior to assessing Version 1 of the process map. The researcher addressed questions surrounding the process map, with reference to a glossary defining individual events. Participants only commented on aspects of the process of which they had direct experience. Appraisal of Version 1 of the process map was undertaken individually. Participants annotated the process map to provide comments on its accuracy and suggested additional steps they believed to be core to the admission process and those to remove. Comments were annotated onto physical process maps. Two authors (JH, DZK) tabulated the comments into a spreadsheet. Comments were independently reviewed and accepted or rejected them. Disagreements were then discussed to achieve consensus, with adjudication from the senior author (HJM) in cases which did not achieve consensus. Accepted comments informed redesign of the Version 1 process map to produce Version 2.

### 2.3 Process map validation

Version 2 of the process map was then modelled with patient data. A retrospective EHR review of 50 consecutive patients admitted for resection of a pituitary tumour was performed. Patients were identified from a prospectively collected database, excluding patients with initial referral under the age of 18 or patients initially referred to the service prior to establishment of the EHR, as critical data was absent in these cases. Patients included for validation were selected from May 2021 – August 2021. Consecutive patients were selected from this period to ensure our cohort reflected contemporary practice in our institution, but also incorporated enough time to ensure adequate follow-up to assess the post-operative pathway. Each admission was reviewed, and clinical events were recorded in sequence. The index admission for each patient was modelled by following the clinical events in sequence through each process map, with a view to identifying deviations between the real-world dataset and Version 2 process map. Event sequences were coded as 1 if in agreement with the process map and 0 if not. Total level of agreement was presented as a percentage of agreement for each patient, and a total average produced for the cohort.

### 2.4 Event analysis

The frequency and reliability of EHR events were then assessed to determine the commonest events occurring in the sample. Events which were present at least 40 times (meaning the event would be present in at least 90% of the total cohort if present only once) were included. A final process map with critical datapoints was produced to indicate event presence grouped by frequency (100%, 90 – 99% and 80 – 89%). We derived the initial 90% cut-off of event presence, and subsequent categories between 80 – 100% based upon previous process mapping research identifying a threshold of 80% as a routine process step (24).

## 3 Results

### 3.1 Process map development

The first iteration of the patient pathway was produced by the authorship (Version 1). Eighteen stakeholders were consulted to redesign the Version 1 process map (**Table 1**). Individuals from neurosurgery (N = 7, 43.8%), endocrinology (N = 5, 36.0%) and ophthalmology (N = 2, 12.5%) dominated the cohort. Most were either consultant or senior trainee doctors (N = 12, 75.0%). The cohort totaled 106 years of experience in managing patients with pituitary adenoma undergoing surgery, with all stakeholders agreeing or strongly agreeing that they were involved in the patient pathway (**Table 1**). 159 individual comments were tabulated from process map annotations. Consensus was achieved for acceptance or rejection in 146 cases (91.8%), with 13 (8.20%) requiring adjudication from the senior author. 94 (59.1%) comments were incorporated into process map redesign and 65 (40.9%) comments were rejected.

**Table 1:** Stakeholder characteristics.

Speciality (N= 18)	
*Neurosurgery*	7 (43.8%)
*Endocrinology*	5 (36.0%)
*Ophthalmology*	2 (12.5%)
*Radiation oncology*	1 (6.25%)
*Pathology*	1 (6.25%)
*Anaesthetic*	1 (6.25%)
*Administrative*	1 (6.25%)
Position (N = 18)	
Professor/Consultant	7
Senior trainee	5
Junior trainee	2
MDT coordinator	1
Clinical Nurse Specialist	3
Patient pathway experience	
Cohort total in years	106
Median in years (IQR)	5 (2 – 10)
Questionnaire responses, median score (IQR)	
I am routinely involved in the patient pathway of pituitary adenoma patients undergoing surgery	5 (4 – 5)
I am directly involved in the patient pathway prior to admission for surgery	4 (2.75 – 4.75)
I am directly involved in the patient pathway during their inpatient stay	4 (4 – 5)
I am directly involved in the patient pathway in the outpatient setting after surgery	5 (4 – 5)

### 3.2 Pituitary patient pathway

Emergencies were admitted immediately, and all other cases were discussed in the pituitary MDT or an outpatient clinic initially. Patients would then attend a pre-operative surgical clinic before admission to hospital. Optional process steps before admission include a separate consent clinic, neuro-ophthalmology review, pre-assessment clinic and pre-operative imaging. Patients are then admitted to the ward and are reviewed by the surgical and anaesthetic teams. If pre-operative imaging is needed, and not yet performed, it would be performed on admission.

After surgery, patients are transferred to recovery, high dependency unit (HDU)/intensive care unit (ICU) or to the surgical ward. Surgical, endocrinology and pituitary CNS reviews occur daily, with in-person endocrinology reviews occurring twice a week, to assess the patient until medically fit for discharge. Some patients will be reviewed by ophthalmology during their inpatient stay.

In the outpatient setting, pathology results are discussed by the MDT. Patients receive follow-up by the pituitary CNS. A blood test may be performed in this clinic, or by their general practitioner, dependent on patient preference and post-code. The patient will then receive appropriate follow-up, directed by their diagnosis (Neurosurgical, endocrinology, ophthalmology, oncology, pituitary CNS). Surveillance imaging and blood tests are completed at defined intervals (according to diagnosis), with review by the pituitary MDT. This process repeats unless the patient is deemed suitable for discharge from the service.

### 3.3 Real-world dataset

#### 3.3.1 Population characteristics

**Table 2** presents the characteristics of the real-world dataset. 50 patients were included with a median age of 55 and a male preponderance (28:22, 56.0%). Most patients had macroadenomas (N = 42, 84.0%) with the most common diagnosis non-functioning adenoma (N = 38, 76.0%). 20 patients (40.0%) had pre-operative visual field defects attributed to their lesion, 15 (30.0%) had anterior pituitary deficits requiring steroid replacement and one patient had a pre-operative posterior pituitary deficit requiring treatment. The majority of patients underwent microsurgical excision of their adenoma (N = 33, 66.0%), with the remainder undergoing endoscopic resection (N = 17, 34.0%). 16 patients (34.0%) had reported complications, of which there were 18. The commonest post-operative complications were electrolyte abnormalities (N = 12), with patients admitted to hospital for a median 4 days.

**Table 2:** Patient population characteristics.

Variable (N = 50)	
Median age in years (IQR)	55 (36 – 69)
M:F ratio (% male)	28: 22 (56.0%)
Tumour size (%)	
* Macro*	47 (94%)
* Micro*	3 (6%)
Diagnosis (%)	
* Non-functioning*	38 (76%)
* Cushing’s disease*	4 (8%)
* Acromegaly*	5 (10%)
* Prolactinoma*	3 (6%)
Pre-operative deficits (%)	
* Visual field defect*	20 (40%)
* Anterior pituitary deficit*	15 (30%)
* Posterior pituitary deficit*	1 (2%)
Surgical approach (%)	
* Endoscopic*	17 (34%)
* Microscopic*	33 (66%)
Median length of stay in days (IQR)	4 (4 –5)

#### 3.3.2 Process map validation

Six patients were excluded from analysis due to critical data losses. 1439 individual events were recorded across the cohort, with 84 discrete event categories identified (**Appendix B**). A mean percentage of agreement from the 44 patients analysed was 92.3% indicating agreement between the final process maps and real-world dataset.

#### 3.3.3 Structured data entry targets

**Figure 2:**
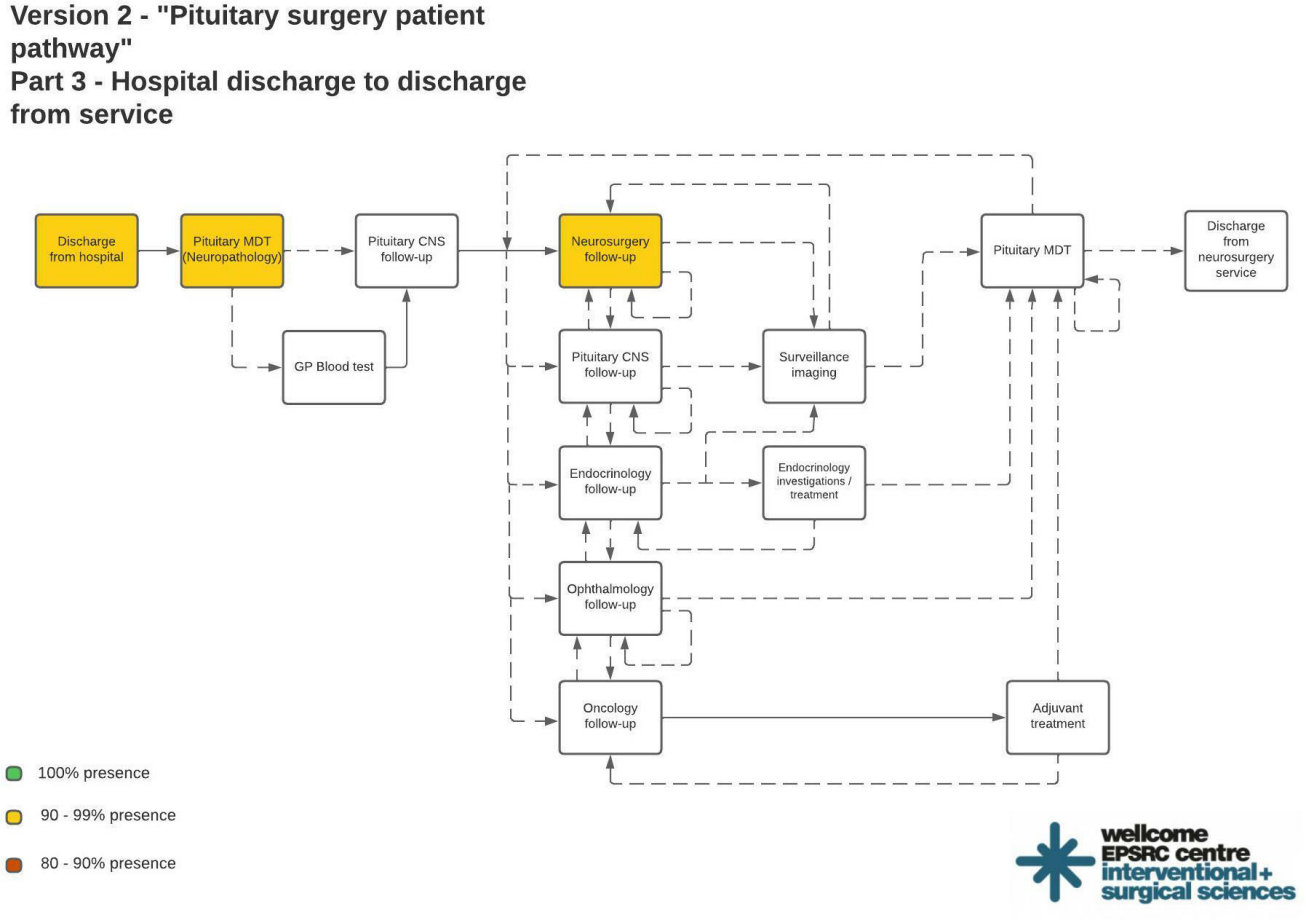
Final process maps indicating most reliable areas of documentation suitable for structured data entry interventions. Color coded process steps indicate % presence in the real world dataset: Green = 100%; Yellow = 90 - 99%; Red = 80 – 90%.

**Figure 2** shows the final process maps indicating reliable targets for structured data entry interventions. Out of 1439 events recorded in the real-world dataset, the commonest events included neurosurgical ward round entries (N = 212/1439, 14.7%), pituitary CNS ward round entries (N = 101/1439, 7.02%), pituitary MDT outcome reports for treatment decisions (N = 88/1439, 6.12%), and pre-operative neurosurgery clinic letters (N = 79/1439, 5.49%). An operation note and neurosurgical ward round entry were present for every patient (**Figure 2**). 43/44 (97.7%) patients had a pre-operative pituitary MDT entry, a pre-operative neurosurgery clinic letter, a post-operative neurosurgery clinic letter, an admission clerking entry, a discharge summary, and a post-operative pituitary MDT with histopathology results.

## 4 Discussion

### 4.1 Principal findings

This is the first application of process mapping to analyse the patient pathway for pituitary surgery, using the highest volume pituitary centre in the United Kingdom as an exemplar. It also advances the application of process mapping through validation with real-world quantitative data to enhance process map validity. Further, we employed the framework set out by Antonacci (20) to guide process map development, which is a first for both surgical and neurosurgical process analysis. This is a strength as such a framework reduces methodological heterogeneity of studies employing process mapping, improving their comparability and critical appraisal. Future process mapping studies should follow this precedent.

The process maps allowed analysis of the key events in the patient pathway required for a patient to undergo surgery for a pituitary adenoma. Matching EHR entries to these events elucidates areas amenable to focus interventions looking to improve data entry. Examination of individual patient records revealed EHR entries which were present in all patient records, suggesting these areas are reliable targets for structured data entry. These included operation notes and neurosurgical ward round entries, which are likely due to the dependency on these entries to inform key stakeholders about the immediate management decisions for this patient group. This finding strengthens the argument for structuring operation notes and ward round entries to improve the quality of documentation, but also offers an opportunity to organise data entry into a format feasible for streamlined automated extraction. We argue that templates for these areas of documentation which include key clinical data points will improve the quality of data entry to the system by ensuring clinicians consider and document important aspects of the patient’s care (for example, presence or absence of intraoperative complications such as carotid artery injury). This will reduce the heterogeneity of data collected and entered, which may indeed result in more standardised healthcare delivery.

The process maps identified areas lacking in adherence to expected documentation standards, presenting targets for future quality improvement efforts. These included admission clerking notes and discharge summaries, which were present in 97% of patients but are expected for every admission. Further, not all patients were reviewed by endocrinology or the pituitary CNS. Discharge summaries, for example, have demonstrated value as data sources in clinical neuroscience research (25, 26), supporting their utility in automated data collection efforts. Yet, concomitant efforts to ensure their completion are important to maintain data integrity. There are several examples of automated data collection systems underperforming, some of which were directly attributed to data input (27), emphasising the value of studying and understanding the process of data collection prior to designing and delivering interventions. This is because automation can be inferior to manual data collection if data entry is inaccurate or incomplete (15, 28), likely resulting in clinical and cost implications if used for decision-making. Acknowledging the limitations of data entering a system is fundamental when assessing performance of an automated system. We found employment of process mapping a useful initial step to begin this process. Our next step is to produce standardized templates for structured data entry and measure their impact on data accuracy and patient care, whilst moving towards clinical data more amenable to automation.

### 4.2 Findings in the context of the literature

As datasets grow the collection and management of data becomes an increasingly resource-intensive task, spurring efforts toward automating data collection. Linked EHR data collection has been shown to be useful in recording basic factual information, demonstrating efficiency gains from automation in infection control (9), but with utility limited to basic datapoints. This means automated data collection for data points of greater complexity, requiring interpretation, may not be feasible. It is these datapoints which require clinical judgement or further analysis which challenge automation.

Baker et al. recognised their automated approach failed to identify exclusion criteria documented in patients notes, influencing the efficacy of their performance review for patients with heart failure (27). The challenge of free-text analysis is beginning to be addressed through natural language processing software, however, there are distinct challenges limiting its success (29). We propose an alternative solution whereby data entry is optimised to permit extraction of these more complex variables, specifically through structured data-entry at critical patient pathway point. This comprises of three core components: (i) identification of the critical areas of documentation to produce structured data entry templates, (ii) production of a core dataset of variables desired and (iii) behavioural interventions for frontline stakeholders involved in data entry to promote uptake and adherence to data entry practices. This study addresses the first of those three components through the application of process mapping, used to produce a shared understanding of our targeted patient pathway. It provided salient targets for interventions aiming to automate data collection.

Patients with a pituitary adenoma undergoing surgery require input from medical and surgical specialties over a significant time-period, meaning their overall patient journey is likely protracted and complex. We found that combining these stakeholder perspectives was an important first step in producing a structured and validated patient pathway to work from. However, we used this patient group as an exemplar, and the methods described could be applied outside of neurosurgery to support automated data collection.

More generally, we recognise that whilst our patient pathway only represents the processes in our centre, it could be used for comparative analyses with other centres for quality improvement initiatives (30). Pituitary surgery is a centralised service in the UK with movement towards centres of excellence globally, meaning fewer neurosurgical centres provide greater shares of pituitary surgical services (31). Therefore, opportunities arise to interrogate the effectiveness and efficiency of healthcare processes themselves. We would advocate pituitary centres to assess and publish their patient pathways to inform the debate on optimising service organisation and delivery, aiming to work towards a nationally standardised care pathway for pituitary surgery. Our methodology of process mapping has been put forward as a critical tool to evaluate neurosurgical processes (30, 32), which can be supplemented with targeted quality improvement strategies to devise strategies for change in neurosurgery and medicine more widely (33).

### 4.3 Strengths and limitations

To the best of our knowledge, this study is the first to produce process maps of a patient pathway using a published framework to guide process map development (20). We supplemented its development with a cross-validation of a real-world clinical dataset to ensure the final process maps represented clinical practice in our institution. Our stakeholder sample included a variety of professions and specialities, ensuring the spectrum of perspectives important to pituitary surgery were included. Process mapping identified critical data points providing salient targets for quality improvement interventions to support our primary aim of automating routine clinical data collection, yet the process map development provides a wealth of opportunities for quality improvement in this patient group. We also identified areas for improvement in adherence to documentation standards for future quality improvement.

Our study also has several limitations. The process maps apply to the processes at our institution and have limited application nationally and internationally, warranting centers to replicate our methods to support comparison and identification of best practices. The use of purposive snow-ball sampling provides a selection bias in identifying frontline stakeholders to provide input to the process maps. We conducted our study and validated the process maps after changes secondary to the COVID-19 pandemic, meaning processes assessed may not be reflective of traditional practice, such as loss of joint clinics between neurosurgery and endocrinology. Further, heterogeneous indications for surgery, such as remission for Cushing’s compared to visual loss for macroadenomas mean nuances in the specific patient pathways may not be captured in our process maps. Finally, our sample size meant differences between subgroups were not analysed.

## 5 Conclusion

This study provides a process map depicting the patient pathway for patients undergoing surgery for a pituitary adenoma in the largest pituitary surgery centre in the UK. This allowed identification of critical points of documentation reflecting key areas in the EHR. These areas will support future quality improvement measures addressing documentation and data extraction to permit automated data collection. We also identified areas of documentation lacking in adherence, providing targets for future quality improvement efforts. In doing so, we have presented our patient pathway for pituitary adenoma surgery which can form the basis for system improvement and redesign, coupled with comparison amongst centres nationally to move towards best practice in this patient pathway.

## Data availability statement

The raw data supporting the conclusions of this article will be made available by the authors, without undue reservation.

## Ethics statement

The studies involving humans were approved by University College London Hospitals NHS Foundation Trust. The studies were conducted in accordance with the local legislation and institutional requirements. The participants provided their written informed consent to participate in this study.

## Author contributions

JH led the study design, data collection and drafted the manuscript. AC supported study design and manuscript editing. DK collected data and edited the manuscript. JF, SW supported study design and data collection. ND edited and approved the final manuscript. SB supported study design and edited the final manuscript. HM was senior author, supported study designed and edited the final manuscript. All authors contributed to the article and approved the submitted version.
