# Supplementary material for: Process analysis of the patient pathway for automated data collection: an exemplar using pituitary surgery
Source: Front Endocrinol (Lausanne). 2024 Jan 12;14:1188870. doi: 10.3389/fendo.2023.1188870 (PMC10811105; doi:10.3389/fendo.2023.1188870)
Supplement: Supplementary file 1 [file DataSheet_1.docx]

Questionnaire

Process mapping the patient pathway for patients undergoing resection of a pituitary lesion.

Lead researcher: John Hanrahan

Email: j.hanrahan@ucl.ac.uk

Version 1

Consent to Participate

**Study title:** Digital workflow analysis of the patient pathway in a pituitary surgical service.

**Study aim**: Elicit the patient pathway of pituitary adenoma patients undergoing surgery to target digital interventions.

**Confidentiality:** Your participation is voluntary and the information you provide, should you wish to do so, will be used for the purpose of refining the content of the assessment tool. Please signal to the researcher any confidential information that you would not want to appear in published form and/or where you wish the source to be made anonymous, please do not hesitate to request so. Should you request anonymity, all identifiable data gathered from this project will remain stored with, and accessible only to the researchers listed.

Authorisation to proceed:

**Print name and add date of consent** (sign separately, below)

I.................................................................................................. Date: ....................................

In line with the GDPR guidelines that came into force on May 25^th^, 2018, I hereby agree to:

| **Item** | **Initial each that you agree to and leave blank if you do not consent to items** |
| --- | --- |
| Be anonymously quoted in publications |  |
| Have my anonymised data used in publications |  |
| Have my anonymised data used in future research |  |
| Be contacted about this research in the future |  |

**Participant signature** (giving consent to participate): ...........................................................

| **Process mapping questionnaire** |
| --- |
| **Instructions** |

**This questionnaire asks for information about your professional background and your involvement with the pituitary patient pathway for those undergoing surgery. It will take about 5 minutes to complete.**

**If you do not wish to answer a question, or if a question does not apply to you, you may leave your answer blank.**

**SECTION A: Professional background**

1. **What is your primary work area or unit in this hospital?**

| 🞏 | a. Neurosurgery |  |  |
| --- | --- | --- | --- |
| 🞏 | b. Endocrinology | |  |
| 🞏 | c. Pathology | |  |
| 🞏 | d. Ophthalmology | |  |
| 🞏 | e. Radiology | |  |
| 🞏 | f. Anaesthesia | |  |
| 🞏 | g. Other (please specify) ______________________ | |  |

1. **What is your job title? ____________________**

**SECTION B: Process experience**

1. **How many years of experience do you in have in managing this patient group? ____________________**

**Please indicate your agreement or disagreement with the following statements.**

|  | **Strongly Disagree** ⯆ | **Disagree** ⯆ | **Neither** ⯆ | **Agree** ⯆ | **Strongly Agree** ⯆ |
| --- | --- | --- | --- | --- | --- |
| 1. I am routinely involved in the patient pathway of pituitary adenoma patients undergoing surgery | 🞎1 | 🞎2 | 🞏3 | 🞎4 | 🞏5 |
| 2. I am directly involved in the patient pathway prior to admission for surgery | 🞎1 | 🞎2 | 🞏3 | 🞎4 | 🞏5 |
| 3. I am directly involved in the patient pathway during their inpatient stay for surgery | 🞎1 | 🞎2 | 🞏3 | 🞎4 | 🞏5 |
| 4. I am directly involved in the patient pathway in the outpatient setting after they have undergone surgery | 🞎1 | 🞎2 | 🞏3 | 🞎4 | 🞏5 |
|  |  |  |  |  |  |

**SECTION C: Initial process map (Please refer to process map now)**

The process map presented to you is an initial process map of the patient pathway of pituitary adenoma patients undergoing surgery at Queen Square from referral to discharge from service. This has been created by two authors based upon their experience.

In this study we want you, as a stakeholder in the process, to help refine this initial process map towards a true representation of the pituitary patient pathway. We appreciate there will be nuances, however, this aims to reflect the pathway as accurately as possible.

This will involve you annotating a physical version of the initial process map, adding or removing steps to reflect your perspective on the patient pathway. If there is an aspect of the process you are not involved with, please highlight this to the researcher. The researcher will work with you through the process map and can clarify any queries.

Your input will be collated and synthesised by the research team to develop a refined process map, which aims to be a better representation of the true patient pathway. We will circulate the refined process map back to you with a justification of changes to validate them.

**SECTION D: Process map feedback**

Thank you for reviewing our process map. Your views will be synthesized with the remaining stakeholder perspectives. The variety of stakeholders include neurosurgeons, endocrinologists, radiologists, neuropathologists, ophthalmologists, pituitary clinical nurse specialists and the MDT coordinator.

**Please indicate your agreement or disagreement with the following statements.**

|  | **Strongly Disagree** ⯆ | **Disagree** ⯆ | **Neither** ⯆ | **Agree** ⯆ | **Strongly Agree** ⯆ |
| --- | --- | --- | --- | --- | --- |
| 1. The overall process is reflected accurately | 🞎1 | 🞎2 | 🞏3 | 🞎4 | 🞏5 |
| 2. The process from presentation to hospital admission is accurate | 🞎1 | 🞎2 | 🞏3 | 🞎4 | 🞏5 |
| 3. The process from hospital admission to discharge from hospital is accurate | 🞎1 | 🞎2 | 🞏3 | 🞎4 | 🞏5 |
| 4. The process from discharge from hospital to discharge from service is accurate | 🞎1 | 🞎2 | 🞏3 | 🞎4 | 🞏5 |
| 5. The appropriate variety of stakeholders have been considered for this project (See text above) | 🞎1 | 🞎2 | 🞏3 | 🞎4 | 🞏5 |

**SECTION E: Proposed interventions**

The intended output of this study is to identify mandatory events in the admission of a patient undergoing surgery for a pituitary tumour. We aim to design digital interventions in Epic (Such as smartphrases) to structure how data is entered at a selection of these mandatory events. We are referring to these interventions as “Structured data entry”.

**Please indicate your agreement or disagreement with the following statements.**

|  | **Strongly Disagree** ⯆ | **Disagree** ⯆ | **Neither** ⯆ | **Agree** ⯆ | **Strongly Agree** ⯆ |
| --- | --- | --- | --- | --- | --- |
| 1. I would be happy to alter my data entry practices supporting structured data entry (e.g. operation note/ward round note template) | 🞎1 | 🞎2 | 🞏3 | 🞎4 | 🞏5 |
| 2. I think structured data entry would improve data quality entered into Epic | 🞎1 | 🞎2 | 🞏3 | 🞎4 | 🞏5 |
| 3. Structured data entry would improve the care of my patients | 🞎1 | 🞎2 | 🞏3 | 🞎4 | 🞏5 |
| 4. Structured data entry would interfere with my current clinical practice | 🞎1 | 🞎2 | 🞏3 | 🞎4 | 🞏5 |
|  |  |  |  |  |  |

**SECTION F: Your Comments**

**Please write any comments you have about research.**

|  |
| --- |

***THANK YOU FOR COMPLETING THIS SURVEY.***
