# Supplementary material for: Process analysis of the patient pathway for automated data collection: an exemplar using pituitary surgery
Source: Front Endocrinol (Lausanne). 2024 Jan 12;14:1188870. doi: 10.3389/fendo.2023.1188870 (PMC10811105; doi:10.3389/fendo.2023.1188870)
Supplement: Supplementary file 2 [file DataSheet_2.docx]

## Appendix B – Event categories

| **Event** | **Frequency** |
| --- | --- |
| Neurosurgery ward round | 212 |
| Pituitary CNS ward round | 101 |
| Pituitary MDT (Treatment decision) | 88 |
| Neurosurgery clinic (pre-operative) | 79 |
| Surveillance MRI (pre) | 78 |
| Endocrinology ward round | 66 |
| Pituitary CNS clinic (post-operative) | 64 |
| Neurosurgery clinic (post-operative) | 57 |
| Surveillance imaging (post) | 52 |
| Preassessment clinic | 44 |
| Operation | 44 |
| Pituitary MDT (Histopathology) | 43 |
| Discharge summary | 43 |
| Admission clerking | 42 |
| Recovery handover | 38 |
| Pre-operative imaging | 36 |
| Endocrinology clinic (pre-operative) | 36 |
| Post-operative neurosurgery review | 35 |
| Radiotherapy | 29 |
| Anaesthetic review | 27 |
| Endocrinology clinic (post-operative) | 26 |
| Referral from endocrinology to neurosurgery | 25 |
| Pituitary CNS clinic (Pre-operative) | 25 |
| ICU ward round | 19 |
| Ophthalmology clinic (Pre-operative) | 17 |
| Ophthalmology clinic (Post-operative) | 13 |
| Lantreotide injection | 9 |
| Oncology clinic (Post-operative) | 7 |
| Consent clinic | 6 |
| Referral from neurology to neurosurgery | 4 |
| Referral from Ophthalmology to neurosurgery | 4 |
| Transfer to ICU | 4 |
| Ward transfer | 4 |
| Dexamethasone suppression test | 3 |
| Endo CNS clinic (post-operative) | 3 |
| Surgical review | 3 |
| Metyrapone day curve | 3 |
| Emergency referral to neurosurgery | 2 |
| Skull base MDT | 2 |
| Rheumatology clinic | 2 |
| Referral from GP to endocrinology | 2 |
| OGTT | 2 |
| Octreotide challenge | 2 |
| Proton beam review | 2 |
| Endo review | 1 |
| DST | 1 |
| Referral from GP to neurosurgery | 1 |
| Post-op MRI | 1 |
| Rheumatology FU | 1 |
| Gastroenterology referral for pituitary MRI | 1 |
| Audiovestibular medicine review | 1 |
| Post-op NSGY review | 1 |
| Nurse recovery handover | 1 |
| Glucagon stress test | 1 |
| Referral from urology to neurosurgery | 1 |
| GST test | 1 |
| Endo FU | 1 |
| Proton beam planning | 1 |
| Inferior petrosal sampling | 1 |
| Endo CNS clinic | 1 |
| ODST | 1 |
| Proton beam therapy treatment | 1 |
| Referral from medical team to neurosurgery | 1 |
| ICU admission | 1 |
| Referral from ophthalmology to endocrinology | 1 |
| Radiotherapy referral | 1 |
| Referral from rheumatology to endocrinology | 1 |
| Radiotherapy review | 1 |
| Cortisol day curve | 1 |
| Readmission | 1 |
| Skull base clinic | 1 |
| Readmission for hyponatremia | 1 |
| Steroid education | 1 |
| ICU handover | 1 |
| Imaging (radiotherapy) | 1 |
| Referal from endocrinology to neurosurgery | 1 |
| Endo clinic | 1 |
| Referral from endocrinology | 1 |
| A&E referral to neurosurgery | 1 |
| Ophthalmology review (Inpatient) | 1 |
| **Grand Total** | **1439** |

## 
